# Influences of maternal reflective functioning on adolescents’ psychosocial adjustment: The mediating role of adolescent’s reflective functioning

**DOI:** 10.1371/journal.pone.0312350

**Published:** 2024-12-26

**Authors:** MinKyoung Park, Hyunjoo Song

**Affiliations:** 1 Samsung Electronics, Gyeonggi-do, Republic of Korea; 2 Department of Educational Psychology, Seoul Women’s University, Seoul, Republic of Korea; Universite de Lille, FRANCE

## Abstract

This study examines the relationship between maternal reflective functioning and adolescents’ reflective functioning and psychosocial adjustment. In Study 1, The Parental Reflective Functioning Questionnaire for Adolescents (PRFQ-A) and Reflective Functioning Questionnaire for Youth (RFQ-Y), multidimensional scales used to assess reflective functioning in parents and adolescents, respectively, were validated in groups of Korean adolescents and mothers. In the results, the three factors were extracted (non-mentalizing, certainty, interest/curiosity) that were similar to those from the original version of the scale. However, the items comprising the interest/curiosity factor of the Korean validated RFQ-Y differed from the original scale. Both the reliability and validity of the scale were confirmed. Study 2 examined the mediating effects of adolescent reflective function on the relationship between parental reflective functioning and adolescent psychosocial adjustment. The structural equation modeling showed a good fit for all three models (adolescents’ uncertainty/confusion, certainty, and interest/curiosity). However, some mediating effects of adolescents’ reflective function were not confirmed. In conclusion, the results indicate that maternal reflective function influences the adolescents’ psychosocial function through the mediating factor of adolescent reflective function, Unexpectedly, maternal interest/curiosity did not have a significant effect on adolescents’ reflective function and psychosocial adjustment. Based on the results, altering Korean mothers’ parenting attitudes may have a positive effect on adolescents’ psychosocial adjustment. This work highlights that further research is needed to understand parent-child relationships in terms of reflective functioning, taking into account the influence of cultural context.

## Introduction

Reflective functioning (RF), also known as mentalization, is the mental process of internally and externally interpreting other’s behavior based on their mental state, including their own and other’s needs, feelings, beliefs, and intentions [1]. In particular, parental reflective functioning (PRF)—which refers to the parent’s capacity to consider the child’s internal experience, make sense of the child’s behavior, and respond based on an understanding of the child’s mental states [2]—is known to play a crucial role in forming healthy parent-child relationships and supporting the child’s psychosocial adjustment (PAS). RF is a critical mechanism for an individual’s emotional regulation and self-control, and it becomes even more important during adolescence, as this is a period of rapid development and potential turmoil. Therefore, understanding RF in the context of the parent-child relationship is particularly important during adolescence when parental influence remains significant. However, there is still a lack of research on the multidimensional aspects of PRF and its specific elements’ impact on adolescents. This study aims to fill this gap by investigating the effects of mothers’ PRF on their children’s RF and PSA.

## Parental reflective functioning, adolescent reflective

### Functioning, and adolescent psychosocial adjustment

Adolescence is characterized by rapid hormonal and physical changes, with dramatic shifts in identity, self-consciousness, and cognitive flexibility [3, 4]. Another important feature is that group interactions become more complex and social behavior increases [5]. Adolescents remain dependent on their parents but also start to gain more independence and autonomy [6]. Intimate relationships also continue to develop, and the social and cognitive processes required for navigating these situations become increasingly complex [7]. During this period, which is prone to rapid mood swings and psychological and identity confusion, the ability of, RF, a key mechanism for self-regulation, becomes even more important.

Studies on adolescents’ RF have primarily found that deficits and failures in RF are linked to various interpersonal and mental health issues. For instance, RF deficiency has been associated with borderline personality disorder, which is characterized by unstable relationships and intense emotional reactivity [8–10]. It has also been connected to vulnerable narcissistic tendencies [11]. Moreover, RF deficits are correlated with internalizing problems such as anxiety and depression, where adolescents may struggle to reflect on their emotional states, leading to overwhelming distress. Externalizing problems, including behavioral and conduct issues, are also tied to RF deficits, as these adolescents may find it challenging to understand the consequences of their actions and to control impulses in social contexts [11–14].

Conversely, the ability to reflect has been positively associated with adjustment and has been shown to be a protective factor against emotional and behavioral problems. Adolescents’ RF is closely linked to empathy, and these abilities play a crucial role in their emotion regulation [8, 15]. RF has acted as a protective factor against aggression and self-harm [16] and mediated the relationship between insecure disorganized attachment and peer problems [17]. For adolescents with antisocial traits, RF has been shown to act as a protective mechanism against aggression [18].

This ability to reflect, which develops throughout the lifespan, is particularly important during adolescence and is considered a key factor in the successful socio-psychological transition to adulthood [19]. Psychosocial adjustment is a concept that includes both psychological and social components and refers to an individual’s ability to interact with the environment, adapt to stress and challenges, and effectively perform social roles [20]. RF is essential for social adjustment because it allows us to represent causal mental states, distinguish between internal and external realities, infer the mental states of others from behavioral and situational cues, and regulate behavior and emotional experiences [21].

RF develops from early relationships with caregivers. As caregivers’ RF has been identified as a key factor in the development of secure attachment and RF in children [22], RF in parent-child relationships has been the focus of research in this area, primarily with infants and parents. Parents with higher PRF show greater empathy and may be more sensitive to their children’s emotional needs [2]. PRF was also associated with mental health issues such as depression and anxiety, as well as parenting attitudes [23].

However, the parent-child relationship remains essential during childhood and adolescence, and evidence suggests that PRF continues to play an important role in influencing adolescents’ RF and overall adjustment. Studies examining the relationship between PRF and adolescents’ RF have found that PRF is significantly related to children’s RF and influences adolescents’ positive self-concept, self-confidence, and early adulthood adjustment characteristics, including romantic relationships [24, 25]. Additionally, Child-focused RF of PRF was associated with child attachment [26]. A deficit in PRF predisposes parents with high levels of self-critical perfectionism to rely on intrusive and pressurizing methods, specifically psychologically controlling parenting, which can significantly impact adolescents’ ability to develop mature emotion regulation strategies and overall adjustment [27].

PRF allows parents to understand themselves and their children based on the mental states behind observed behaviors, so poor PRF can lead to emotional turmoil and a variety of negative parenting attitudes and relationship problems. Conversely, reflective parenting can help adolescents understand their feelings and thoughts about the changes they are experiencing in their development [24]. Moreover, in parent-adolescent relationships, where intense emotions are common, parents with high levels of RF may be more sensitive to their child’s emotions and better able to manage their child’s emotional imbalances. This can have a positive impact on their children’s psychosocial adjustment, which includes coping effectively with stressful challenges, forming healthy interpersonal relationships, and maintaining overall mental health.

### Multidimensional assessment of reflective functioning

Fonagy and colleagues have continued to develop self-report RF scales in response to the need for simpler measures of RF. In particular, The Parental Reflective Functioning Questionnaire for Adolescents: PRFQ-A [28] and Reflective Functioning Questionnaire for Youth: RFQ-Y [11] were developed to measure multidimensional aspects of RF.

The PRFQ-A is an adaptation of the PRFQ [29], which was designed for parents of toddlers aged 0 to 5 years, to make this scale more appropriate for parents of adolescents aged 12–18 years. Based on theories of mentalizing ability, the PRFQ consists of items that aim to capture three main characteristics of RF: 1) interest in and curiosity about mental states, 2) the ability to recognize uncertainty (opacity) in mental states, and 3) characteristics of the pre-mentalizing mode in parents with severe impairments in PRF. The PRFQ-A has not yet been published, but its construct validity has been confirmed [27].

Scales to measure RF in adolescents were first validated with a single-scale 46-item version [8]; additionally, a shortened 8-item version measuring two aspects of mental states, certainty, and uncertainty, was later reported [12, 30]. Meanwhile, Duval et al. [11] factor analyzed the 46-item RFQ-Y and validated a 25-item reflective functioning questionnaire for adolescents consisting of three factors. These subscales were “Uncertainty/Confusion about mental states,” “Interest/Curiosity in mental states,” and “Excessive certainty about mental states of others,” similar to the factor structure of the PRFQ [29]. Each subscale correlated with measures related to personality disorders, such as externalizing and internalizing problems, borderline personality disorder, and narcissistic personality disorder in adolescents, thus proving the RFQ-Y an appropriate tool for assessing reflective functioning.

These two scales have identified three theoretically distinct and clinically meaningful dimensions of RF. This is significant because it expands our understanding of the complexity of RF and the nature of each dimension. Aspects such as IC about mental states can particularly contribute to a clearer understanding of what good mentalization looks like. This provides a foundation not only for recovery from mentalization failure but also for promoting positive mentalization traits. Therefore, it can play a crucial role in interventions such as Mentalization-Based Treatment and parent education that focus on RF.

### The present study

Mothers are often the primary caregivers when raising children, and this is true in Korean society as well. This traditional role of mothers continues into adolescence, and the influence of mothers on their children’s development remains significant. In this context, the present study focuses on the mother-child relationship. In this work, two studies are conducted to explore mother-child relationships and adolescents’ psychological adjustment to RF.

Firstly, Study 1 validates the PRFQ-A [28] and RFQ-Y [11] in Korean populations using an exploratory factor analysis and a confirmatory factor analysis, as well as reliability and criterion validity. Study 2 examines the relationship between maternal PRF, adolescent RF, and psychological adjustment. Specifically, we explore the mediating role of adolescents’ RF in the relationship between maternal PRF and adolescent psychosocial adjustment. In particular, the three subscales of each scale are explored separately to determine the effects of multidimensional aspects of maternal PRF on each aspect of adolescent RF.

## Study 1: Validation of the reflective functioning questionnaire

The purpose of Study 1 was to determine the reliability and validity of the PRFQ-A [28] and RFQ-Y [11] in Korean. Specifically, this study aimed to test the factor structure of these scales to confirm whether the theoretical constructs proposed in previous studies are applicable in the Korean context.

As in previous studies, both scales were assumed to consist of three independent factors. To validate convergent validity, we examined correlations between each scale and both theoretically and empirically related variables. We primarily used variables from the original validation study, supplemented by a few additional variables chosen for their relevance to the constructs. This approach allowed us to maintain consistency with the original study while ensuring the translated scales accurately measured the intended constructs in the Korean context.

Each subscale of the PRFQ-A was expected to be associated with traits such as mentalization failure, empathy, mental health, and parenting attitudes. Specifically, we hypothesized that pre-mentalizing (PM), which reflects vulnerable reflective functioning, would negatively affect mothers’ mental health and parenting attitudes. In a previous study [28], Certainty about Mental States(CMS), a tendency of parents to be overly certain about the mental states of their child, and Interest and Curiosity(IC) in their child’s mental state were associated with positive parenting attitudes but were also related to intrusive parenting attitudes. This supports the notion that high levels of CMS and IC reflect traits of hypermentalizing. In this study, we did not formulate specific hypotheses about CMS and IC but explored the relationships between these concepts and the other scales in an exploratory manner.

For the RFQ-Y, based on the theoretical background, we hypothesized that uncertainty and confusion about mental states (U/C) will positively relate to mentalizing failure, lower empathy, internalizing and externalizing problems as well as personality pathology. In previous research [11], certainty about mental states (Certainty) and interest/curiosity about mental states (I/C) scales have either been unrelated to other scales or have shown static or negative correlations with personality pathology and externalizing problems. Therefore, we explored the relationships between Certainty, I/C, and other scales without making specific assumptions.

## Study1-1: Korean version of the Parental Reflective Functioning Questionnaire for Adolescents (PRFQ-A)

### Methods

#### Participants and procedures

Study 1–1 was conducted with mothers of adolescents aged 12 to 18 years (N = 404). An online research organization collected responses from participants through an online survey from October 23 to 30, 2018. The survey form included a study description and an electronic consent (e-consent) box, which participants must check electronically before beginning the survey. Mothers in the study were aged 33–59 years (M = 44.60, SD = 4.58), with 75% having a college degree, 19.1% a high school diploma, 5.7% a graduate degree or higher, and 2% a junior high school diploma. Overall, 98% were married, and 58.2% were employed (24.8%, part-time, 33.4%, full-time). Additionally, 49.9% of the respondents’ children were in middle school, 41.2% were in high school, 8.2% were in elementary school, and 0.7% were in college. Finally, 47.5% of the children were male, and 52.5% were female. This study received ethical approval from the Seoul Women’s University Institutional Review Board (SWU IRB- 2018A-44).

#### Measures

*Parental Reflective Functioning*. The PRFQ-A [28] was translated into Korean. It consists of three subscales, “’Pre-Mentalizing Modes (PM),” “Certainty about Mental States (CMS),” and “Interest and Curiosity (IC),” and is a 7-point scale with a total of 18 items. In a study using the PRFQ-A [28], the internal consistency values were .62, .78, and .71 for PM, CMS, and IC, respectively.

We adapted the English and Dutch PRFQ-A into Korean for this study on the UCL website (www.ucl.ac.uk) with the authors’ consent. The translation process was English to Korean to English, and the final back-translated items were checked with the original authors. The Dutch version of item 7, “I find it difficult to empathize with the fantasies of my son/daughter,” was deemed more suitable for parents of adolescents, compared to the English version, “I find it hard to actively participate in make-believe play or imaginary activities with my child.” In this case, a discussion with the original authors led to translating from Dutch to English and then to Korean.

*Mentalization*. To explore the relationship between RF and mentalization failure, we used the Korean version of the Mentalization Questionnaire (MZQ**)** [31, 32]. K-MZQ consists of 13 items and assesses four dimensions. The internal consistency for this scale was .91 for the total scale, .80 for refusing self-reflection, .72 for emotional awareness, .78 for psychic equivalence mode, and .72 for regulation of affect.

*Empathy*. The Korean-modified version of the interpersonal reactivity scale [33, 34] was used. From this scale, we specifically assessed perspective-taking (7 items), which measures cognitive empathy, and empathic concern (7 items), which measures emotional empathy, as these two subscales are directly related to the construct of empathy. The internal consistency values were .67 and .71, respectively.

*Attachment*. The shortened Korean version of the Experiences in Close Relationships Questionnaire-Revised (ECR-R) [35, 36] was used in this work. The internal consistency values for anxiety (7 items) and avoidance (7 items) were .94 and .91, respectively.

*Symptomatic distress*. The Patient Health Questionnaire 9-item depression scale (PHQ-9) [37] was used to assess depression, and this scale had an internal consistency of .91. The Generalized Anxiety Disorder-7 (GAD-7) scale [38] was used to measure anxiety, and this scale had an internal consistency of .91.

*Parenting*. The Korean version of the Parents as Social Context Questionnaire (PSCQ) [39, 40] was used to measure parenting style in this work. The PSCQ consists of six scales, totaling 23 items, including “warmth,” “rejection,” “structure,” “chaos,” “autonomy support,” and “coercion.” In this study, the internal consistency values were .72, .80, .57, .79, .79, and .85, respectively, for each scale. The Korean version of the Parenting Sense of Competence (PSOC) scale [41, 42] was used to measure parenting self-efficacy. This scale consists of 13 items, including the cognitive dimensions of parenting self-efficacy (9 items) and emotional dimensions of parenting frustration and anxiety (4 items). The internal consistency values were .85 for parental efficacy and .80 for parental anxiety.

#### Data analysis

The collected data was divided into two independent samples to conduct EFA and CFA. Descriptive statistics were conducted on both samples 1 and 2. An exploratory factor analysis (EFA) was conducted to confirm the factor structure of the PRFQ-A based on the responses of Sample 1 (N = 203). Principal axis analysis and Promax rotation were used as the primary estimators for the factor extraction and rotation. Additionally, a parallel analysis was conducted to estimate the number of factors more rigorously. Specifically, parallel analysis is a method that linearly compares the identified eigenvalues with the eigenvalues obtained from a randomly generated sample [43]. This method is more theoretically sound than other procedures that estimate the number of factors based on eigenvalues (Kaiser’s method, screening test) and uses empirical evaluation to provide a more accurate estimate of the number of factors [44]. For the selection of factor items, we excluded items with factor loadings of .40 or lower, following the rationale for item deletion outlined by Pett et al. [45]. Next, confirmatory factor analysis (CFA) was conducted based on the responses of Sample 2 (N = 201). We used CMIN/DF, which stands for the ratio of the chi-square value (CMIN) to the degrees of freedom(DF), the Tucker-Lewis index (TLI), the comparative fit index (CFI), the root mean square error of approximation (RMSEA), and Standardized Root Mean Square Residual (SRMR), as fit indices. CMIN/DF is generally considered a good fit if it is below 2. TLI and CFI are generally regarded as excellent models if they are above .90. For RMSEA, a value of .05 to .08 is usually considered to indicate an adequate model, and a value below .05 is considered to indicate a good model [46]. SRMR values of 0.08 or less indicate a ’good’ fit, and lower values suggest a model that better fits the data. [47]. To assess the internal consistency of the scores, Cronbach’s α values were calculated for each factor identified through EFA and CFA. Finally, a correlation analysis was conducted with variables related to the PRFQ-A to examine the relationships between the identified factors and other relevant variables. This analysis provided evidence of the internal structure of the PRFQ-A and its relationships with external variables. The data analysis was conducted with SPSS version 22.0 and AMOS version 23.0.

### Results

#### Factor structure of the K-PRFQ-A

Descriptive statistical analysis results indicated that the skewness and kurtosis for each question in both Sample 1 and Sample 2 did not exceed absolute values of 2 and 7, respectively, indicating that the normality assumption was satisfied [48] (see S1 and S2 Tables). To confirm the factor structure of the PRFQ-A, parallel analysis and EFA were conducted. The KMO value was .868, and Bartlett’s test of sphericity was also significant (p < .01), indicating that the data were suitable for factor analysis. Three factors with eigenvalues of 1 or greater were identified, and the parallel analysis confirmed that the three factors were appropriate. After excluding item 1, which had a factor loading of less than .3, 17 items were assigned to the three factors, and the three factors explained 64.77% of the total variance (S3 Table).

The three factors were as follows: Pre-Mentalizing Modes (PM); Certainty about Mental States (CMS); and Interest and Curiosity (IC). However, some items were assigned differently from the original PRFQ [29]. Specifically, item 18(“I believe there is no point in trying to guess what my child feels”) is reverse-scored. In the original scale, this item belonged to the I/C. However, in this study, it was assigned to PM with a value of -.756. Considering the nature of the reverse-scored item, the original scale viewed efforts to guess what a child is feeling as reflecting an interest and curiosity about the child’s mental state. However, the literal meaning of the item implies an attitude of not trying to guess the child’s feelings or believing it is pointless, which indicates a lack of mentalization or a rejection of reflection. Therefore, in the Korean version of the Parental Reflective Functioning Scale, it was deemed appropriate to include item 18 in the PM without reverse scoring.

**Fig 1:**
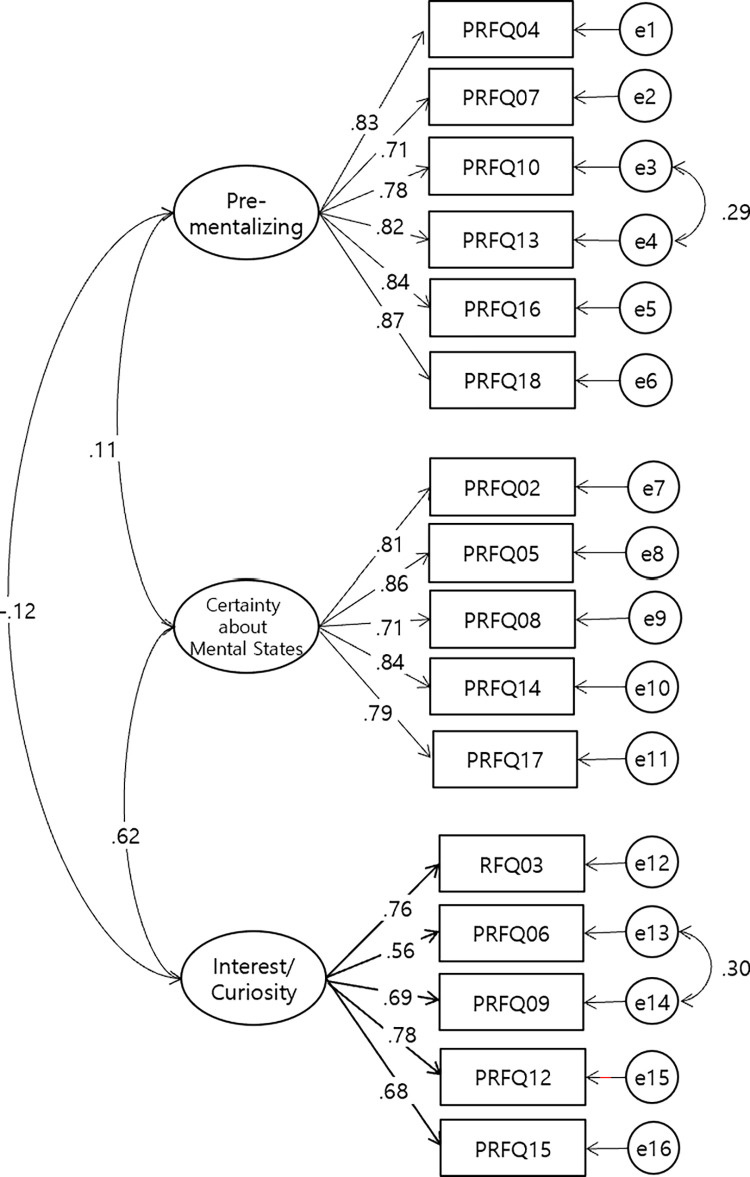
CFA in K-PRFQ-A. Note: The final model excludes items 1 and 11. Rectangles indicate measured variables, ellipses represent latent constructs, and circles represent residuals, with lines between residuals representing correlations. All standardized regression weights for the factor loadings were statistically significant (p < .001).

Next, We conducted a confirmatory factor analysis(CFA) using maximum likelihood to evaluate the fit. After excluding item 11, which had a low factor loading of .044, we reanalyzed 16 items. The model fit indices were as follows: CMIN = 211.919 (df = 101, p < .001), CMIN/DF = 2.1972, TLI = .925, and CFI = .936, RMSEA = .077, SRMR = .0731. Based on the modification index (MI), the measurement model was modified to improve the fit by setting the covariance between the measurement errors of the items that shared similar characteristics among the items included in each factor. The revised goodness-of-fit index (CMIN/DF), which was the value of x^2^ (195.682) divided by the number of degrees of freedom (99), was 1.977. CFI = .949, TLI = .938, and RMSEA = .069, SRMR = .0689 were all acceptable [Fig 1]. The internal consistency values were .92, .90, and .84 for PM, CMS, and IC, respectively.

*Relationships with Mentalization and Empathy*. PM was not correlated with CMS and IC, but CMS and IC were positively correlated with each other (S4 Table). PM was positively correlated with mentalizing difficulties and its subscales, but negatively correlated with empathic consideration and perspective-taking. CMS was positively correlated with empathic consideration and perspective-taking, and IC was positively correlated with empathic consideration and perspective-taking. Relationships with attachment and symptomatic distress.

*Relationships with Attachment and Symptomatic Distress*. In this work, PM was positively correlated with attachment avoidance and attachment anxiety (S5 Table). In contrast, CMS and IC were not related to attachment anxiety, and CMS and IC were negatively correlated with attachment avoidance. Regarding symptomatic distress, PM was statically related to both depression and anxiety.

*Relationships with Parenting*. PM was positively correlated with rejection, confusion, and coercion, but negatively correlated with warmth, autonomy support, and parenting self-efficacy (S6 Table). In contrast, CMS showed negative correlations with rejection and coercion, and positive correlations with warmth, autonomy support, and parenting self-efficacy. Similarly, IC was negatively correlated with rejection, confusion, and coercion, while positively correlated with warmth, autonomy support, structure, and parenting self-efficacy.

## Study1-2: Validation of the Korean version of the Reflective Functioning Questionnaire for Youth (RFQ-Y)

### Methods

#### Participants and procedures

Overall, 496 respondents participated in this part of the study. Data was collected via an online survey by an online research organization from November 13–17, 2018. The survey form included a study description and an electronic consent box to begin the survey. For minors under 18 years of age, we also obtained parental consent for them to participate in the study. To confirm parental consent, the parents of participants were asked to provide details about their relationship to the participant, their full name, cell phone number, and email address, and they were informed about privacy.

The participants’ ages ranged from 13–21 years old (M = 17.66, SD = 2.76), and 52.2% were male and 47.8% female. Additionally, 28.8% were middle school students, 25.8% were high school students, 44.4% were college students, and 1.0% were other. This study received ethical approval from the Seoul Women’s University Institutional Review Board (SWU IRB-2018A-80).

#### Measures

*Reflective Functioning*. The Reflective Functioning Questionnaire for Youth (RFQ-Y) [11] was adapted and used in Korean. The RFQ-Y consists of three subscales, including “uncertainty/confusion about mental states” (U/C), “excessive certainty about mental states of others” (Certainty), and “interest/curiosity in mental states” (I/C). It is a 25-item 6-point rating scale, and the internal consistency values for each scale were .89, .80, and .75, respectively. Translation was performed in the order of English to Korean to English with the consent of the corresponding author of the RFQ-Y (46 items) [8]. The final back-translated items were checked with the original author.

*Mentalization*. The K-MZQ [32] was used to assess mentalization. The internal consistency of the total scale in this study was .88, and the internal consistency for refusing self-reflection was .74, for emotional awareness was .66, for psychic equivalence mode was .74, and for regulation of affect was .64.

*Empathy*. The Korean version of the Empathy Quotient Short Form (EQ-Short-K) [49], developed by Baron-Cohen and Wheelwright [50] and simplified to 22 items by Wakabayashi et al. [51], was used in this work. The EQ-Short-K comprises a single 11-item construct measuring the cognitive, emotional, and social aspects of empathy. This scale had an internal consistency of .88 in this study.

*Internalizing and Externalizing Problems*. PHQ-9 [37] for depression and GAD-7 [38] for anxiety were used to examine the relationship between RF and internalizing problems. The internal consistency values were found to be .91 for the PHQ-9 and .92 for the GAD-7. To explore the relationship between RF and externalizing problems, two subscales of the Executive Function Difficulty Questionnaire for Children and Adolescents [52] related to reflective functioning, including behavioral control difficulties (11) and emotional control difficulties (8), were used. The internal consistency values for these subscales were .90 and .91, respectively.

*Borderline traits*. The Korean version of the Personality Assessment Inventory-Borderline Feature Scale (PAI- BOR) [53], one of the 11 clinical scales of the Personality Assessment Inventory (PAI) [54], was used. This scale consists of 24 items related to emotional instability, identity problems, negative relationships, and self-damage, which are core features of borderline personality disorder. The Korean version consists of 23 items, as one was removed due to a low internal correlation between the item-total scores. In this study, 18 items were used after excluding 5 items with low item-total score reliability, and the internal consistency of the scale was .94.

*Narcissism traits*. The Korean version of the Pathological Narcissism Inventory (PNI) [55, 56] was used. The Korean version consists of six factors, excluding self-hiding. Exploitation, grandiose fantasies, and self-sacrifice-self-catheterization are categorized as relating to grandiosity, and fluctuating self-esteem, devaluation, and privilege anger are classified as relating to vulnerability. This internal consistency was .97 overall in this study, and the internal consistency values for the scales were .93 for grandiosity, and .96 for vulnerability.

#### Data analysis

The collected data was divided into two independent samples for conducting EFA and CFA. Descriptive statistics were conducted on both samples 1 and 2. The initial data from Sample 1 (N = 257), collected through an online survey created by the researcher and a research organization, were used for the EFA. Principal axis analysis and Promax rotation were used, and a parallel analysis was conducted to estimate the number of factors. For the selection of the factor items, we excluded items with a commonality of .25 or less [57], factor loadings of .40 or less [45], and cross-loadings of .15 or less [58] based on theoretical grounds. Next, a CFA was conducted based on Sample 2 (N = 239). The CMIN/DF, Tucker-Lewis index (TLI), comparative fit index (CFI), root mean square error of approximation (RMSEA), and Standardized Root Mean Square Residual (SRMR), were used for the fit indices. Additionally, measurement invariance(MI) was tested using multi-group confirmatory factor analysis (MGCFA) to assess the consistency of the measurement model across groups, focusing on configural, metric, scalar, and residual invariance, including gender. To assess the internal consistency of the scores, Cronbach’s α values were calculated for each factor identified through EFA and CFA. Finally, a correlation analysis was conducted with variables related to the RFQ-Y to examine the relationships between the identified factors and other relevant variables. SPSS 22.0 and AMOS 23.0 were used to analyze the data.

### Results

*Factor Structure of the K-RFQ-Y*. Descriptive statistical analysis results showed that, for both Samples 1 and 2, the skewness and kurtosis of each question did not exceed absolute values of 2 and 7, respectively, indicating that the normality assumption was satisfied (see S7 and S8 Tables). In terms of the results of the EFA, the KMO value was .856, and Bartlett’s test of sphericity was also significant(p < .01), indicating that the data were suitable for factor analysis. The parallel analysis confirmed that three factors were appropriate, so the number was fixed at three. The factor analysis excluded item 2, which had a commonality of .25 or less, and item 17, which had a commonality of .4 or less. Three items with cross-loadings of .1 or less were excluded, and all three items were assigned to Factor 1 (U/C) and Factor 3 (I/C). The Factor 1 and Factor 3 loadings were .335 and .361 for item 1, .410 and .505 for item 4, and .401 and .429 for item 8. A total of 20 items, excluding 5 items, were assigned to the three factors, explaining 50.66% of the total variance (S9 Table).

Each factor was similar to RFQ-Y [11], but some items were assigned to different factors. Item 3 belongs to U/C in the original scale but was assigned to I/C in this study. Item 6 (I believe other people are too confusing to bother figuring out) was reverse-scored to the I/C factor in the original scale but was assigned to U/C (-.506) in this study. This item has a similar meaning to PRFQ item 16 (Often, my child’s behavior is too confusing to bother figuring out), which was assigned to PM in the PRFQ. Therefore, in this scale, it was considered more appropriate to assign item 6 to U/C, without reverse-scoring. Compared to the original factor of Duval et al. [11], Factor 1 was named uncertainty/confusion about mental states (U/C), and Factor 2 was named excessive certainty about mental states of others (Certainty). We named Factor 3 interest/curiosity about the mental states of others (I/C) because the final item is mainly composed of items that reflect interest in the mental states of others.

**Fig 2:**
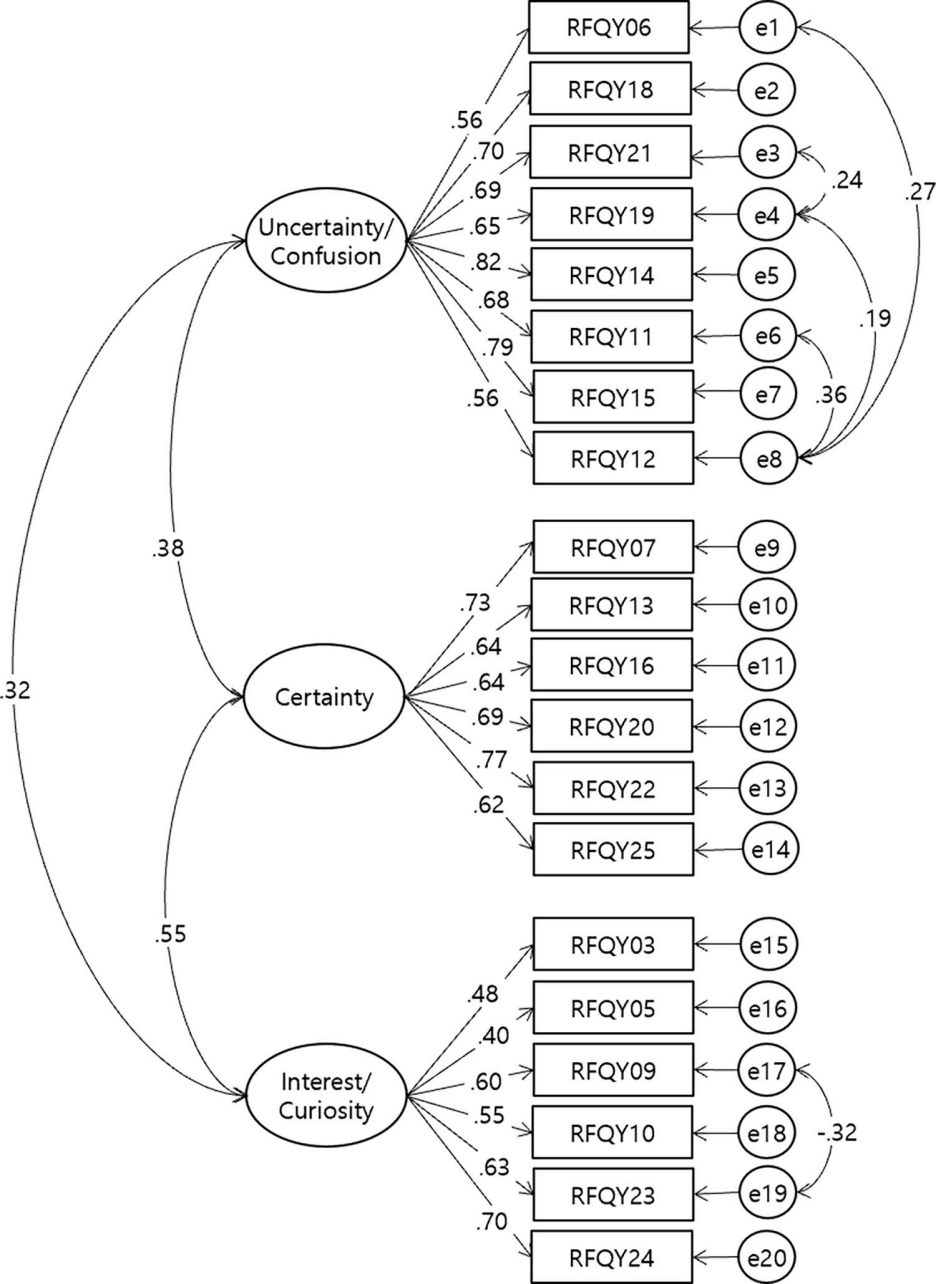
CFA in K-RFQ-Y. Note: The final model excludes items 1, 2, 4, 8, and 17. Rectangles indicate measured variables, ellipses represent latent constructs, and circles indicate residuals, with lines between residuals representing correlations. All standardized regression weights of the factor loadings were statistically significant (p < .001).

Next, we conducted CFA using the maximum likelihood method. The fit indices were as follows: CMIN = 489.24637 (df = 206, p < .001), CMIN/DF = 2.37498, TLI = .858, CFI = .874, RMSEA = .06946, and SRMR = .0646. The measurement model was modified based on the modification index (MI). The CMIN/DF of the revised goodness-of-fit index was 1.866. CFI = .920, TLI = .906, RMSEA = .060, and SRMR = .0624 indicating an overall acceptable fit index [Fig 2]. The MI analysis, including the gender variable, showed that for configural invariance, CFI = .883, TLI = .863, RMSEA = .052, and CMIN/DF = 1.647. For metric invariance, CFI = .878, TLI = .864, RMSEA = .052, and CMIN/DF = 1.642. Scalar invariance yielded CFI = .871, TLI = .865, RMSEA = .052, and CMIN/DF = 1.639, while residual invariance showed CFI = .868, TLI = .872, RMSEA = .050, and CMIN/DF = 1.604. While the RMSEA values were within acceptable ranges, both the CFI and TLI values fell below the .90 threshold, indicating a somewhat insufficient model fit. These results indicate partial support for MI, suggesting that invariance was maintained only under certain conditions. The internal consistency for each scale was .88, .84, and .72 for U/C, Certainty, and IC, respectively. In the Duval et al. [11] study, the internal consistency values were .89, .80, and .75, respectively.

*Relationships with Mentalization and Empathy*. U/C was positively correlated with the MZQ total score, as well as its subscales of mental equivalence, denial of self-reflection, emotional awareness, and emotional regulation (S10 Table), Certainty showed a positive correlation only with denial of self-reflection. Additionally, I/C was positively correlated with the MZQ total score, the subscales of mental equivalence and emotional recognition, and the empathy scale.

*Relationships with Internalizing and Externalizing problems*, *Borderline Traits*, *and Narcissism Traits*. U/C was positively correlated with depression, anxiety, difficulties in controlling behavior and emotions, borderline personality traits, and both narcissistic grandiosity and vulnerability (S11 Table). Certainty also showed positive correlations with borderline personality traits, narcissistic grandiosity, and vulnerability. Finally, I/C was positively correlated with narcissistic grandiosity and vulnerability.

### Discussion

The Korean version of the PRFQ-A comprised 16 items and identified the same three factors as the original scale. The fit indices of each factor were significant. From the PRFQ-A, PM was found to be negatively related to maternal characteristics, such as mentalizing difficulties, empathy, attachment, depressive anxiety, parenting attitudes, and parenting efficacy. This result indicates that PM was not only related to mothers’ maladaptive characteristics but also strongly related to negative parenting attitudes. Conversely, CMS and IC were not related to mothers’ mental health but were correlated with adaptive parenting attitudes. In relation to attachment, PM was positively related to attachment anxiety and avoidance, especially attachment anxiety. This result is consistent with research that suggests that overactivation of the attachment system, or deactivation due to mentalizing inhibition, is associated with mentalizing difficulties [59].

The relationships between CMS, IC, and attachment vary across previous studies. In the PRFQ study [29], IC and CMS were not related to attachment, nor were they related to symptom scales. The researchers suggested that adaptive PRF may not be related to attachment and explained this as a loose coupling between attachment and RF. However, Rostad and Whitaker [60] found that both scales were negatively correlated with low levels of attachment avoidance. In the present study, the CMS and IC scales were negatively correlated with avoidant attachment, suggesting that positive PRF is partly related to attachment.

The Korean version of the RFQ-Y also identified the same three factors as the original scale, and the fit indices for each factor were significant. However, the item composition of each factor differed from the original scale in some respects. This was particularly true for the excluded items because they loaded similarly onto U/C and I/C, suggesting that the two scales should be examined more carefully.

Specifically, the U/C items are appropriate for measuring non-mentalization because they retrospectively assess the circumstances and consequences of a mentalization failure. For example, item 11, “When I get angry, I say things without really knowing why I am saying them,” assesses the experience of failing to mentalize in a situation of anger, resulting in inappropriate behavior. However, the excluded items were questions about confusion regarding one’s own emotions and behaviors (1. “I worry a lot about what people are thinking and feeling,” 4. “I often get confused about what I am feeling,” 8. “I don’t always know why I do what I do”). These items measure confused emotions and behaviors, but they also reflect a meta-perspective of observing confused mental states and behaviors and an interest in knowing mental states, and there seems to be a tendency for responses across both U/C and I/C scales.

While I/C in the original scale refers to interest and curiosity about one’s and others’ mental states, IC in this study primarily includes consideration of others’ perspectives and curiosity and understanding of others’ behavior. The items that focus on the self include the following: 3. “I feel that, if I am not careful, I could get in the way of another person’s life” 24. “I pay attention to the impact of my actions on others’ feelings.” However, these items are concerned with the effects on others.

One possible explanation for the differences in the construction of IC items between the Korean and original scales is related to cultural differences in the motivation to have a reflective attitude. One framework for explaining cultural differences is “individualism-collectivism.” In collectivistic cultures, what others (especially significant others within one’s group) think and expect of a person is more important than what that person thinks, feels, and is interested in; therefore, how well an individual fulfills their role in a relationship and how others view them are essential [61]. Previous studies have reported that Korean family relationships embrace a mix of collectivist familism and individualism [62] and that Korean children and adolescents’ beliefs about family relationships retain much of the traditional familistic culture [63].

Regarding the relationship between the RFQ-Y and the other variables, we first found static correlations between the RFQ-Y subfactors U/C and Certainty. Fonagy et al. [64] suggested that high scores on the Certainty (RFQ-C) scale may be explained by the tendency to over-schematize one’s own and others’ actions or intentions without evidence and that respondents may give biased responses because they perceive themselves to be good at mentalizing. Therefore, individuals with high scores on the Certainty scale may include people who respond with certainty about their actual mental state and people who respond with certainty without sufficient reflection.

All three scales had no or very low correlations with the empathy scale. Notably, previous studies have reported mixed results regarding the relationship between RF and empathy [2, 12, 32, 64]. For example, Park and Song [30] found that RF was significantly related to cognitive empathy but not to integrated empathy, which includes emotional, cognitive, and social empathy. Indeed, in the present study, we used a measure of empathy that includes emotional, cognitive, and social components, which have been confirmed to be distinct characteristics of empathy.

In this work, U/C was related to both mentalizing failures and internalizing and externalizing problems, as well as to borderline and narcissistic traits, thus confirming it as a measure of RF failure, consistent with previous research. Certainty was significantly positively related to narcissistic traits and borderline personality traits. Indeed, this result is consistent with research showing that hypermentalization is associated with borderline personality disorder in adolescents [10, 65].

I/C was weakly positively related to anxiety and borderline personality, as well as to emotion recognition, which reflects difficulty recognizing one’s emotions. Given that the primary focus of I/C is on others’ state of mind and one’s state of mind in relation to others, high I/C may increase an individual’s attention to others’ behaviors and states of mind while inhibiting reflection on their own emotions, thus reflecting a fear of the impact one may have and the consequent judgment of others. In addition to the differences in item structure, I/C has relatively unstable characteristics, as evidenced by its low reliability compared to other scales and low factor loadings in the confirmatory factor analysis. In light of these considerations, further research is needed to better understand the nature of I/C, and careful interpretation is required when using the scale in research and clinical practice. The MI showed that while some invariance was supported with the gender variable, the CFI and TLI values fell below .90, indicating insufficient model fit. This suggests the measurement tool may not work equally across gender groups, requiring caution in interpreting the results. The small and slightly imbalanced samples of boys (N = 128) and girls (N = 111) may have influenced these findings. Future research should recruit larger, more balanced samples to improve the robustness of MI.

## Study 2: The relationship between parental reflective functioning, adolescent reflective functioning, and adolescent psychosocial adjustment

The purpose of Study 2 was to determine the relationship between maternal parental PRF, adolescent RF, and adolescent PSA. PSA was measured by self-efficacy, interpersonal efficacy, and school adjustment (relationships with friends and teachers). Self-efficacy and interpersonal efficacy refer to beliefs and confidence in one’s abilities in the self and interpersonal domains. These are associated with a positive self-concept and are considered important factors in PSA. Adjustment with friends and teachers was used as a primary indicator to assess interpersonal relationship adjustment in the school setting.

First, we expected significant correlations between maternal PRFs (PM, CMS, IC), adolescents’ RFs (U/C, certainty, I/C), and PSA. Next, we hypothesized that adolescents’ RFs (U/C, certainty, I/C) would mediate the relationship between maternal PRFs and PSA, and we analyzed the paths of the three models according to each mediator. Considering prior research, maternal PM was expected to negatively impact adolescents’ RF and PSA. To our knowledge, no previous research has explored the multidimensional relationship between parent and adolescent RF. Therefore, we did not make specific assumptions about the effects of CMS and IC.

### Method

#### Participants and procedures

Study 2 was conducted with a separate group of participants from Study 1. Study 2 included 13- to 18-year-old adolescents and their mothers (176 pairs, 352 participants). Data were collected from November 21–25, 2018, via an online survey conducted by an online research organization. The survey form included a study description and an electronic consent box to be checked before starting the survey. Parental consent for adolescent participation in the study was obtained, and parental information was collected as in Studies 1–2. The mothers’ ages ranged from 32–66 years (M = 46.23, SD = 6.04), and the adolescent’s ages ranged from 13–18 years (M = 15.23, SD = 1.56). Overall, 45.5% of the adolescent participants were male, and 54.5% were female. This study received ethical approval from the Seoul Women’s University Institutional Review Board (SWU IRB-2018A-79).

#### Measures

*Maternal PRF*. The K-PRFQ-A from Study 1–1 was completed by the mothers. The internal consistency values were .93 for PM, .89 for CMS, and .78 for IC.

*Adolescents’ RF*. The K-RFQ-Y from Study 1–2 was used with adolescents. The internal consistency values were .91 for U/C, .89 for Certainty, and .76 for I/C.

*Psychosocial Adjustment*. The following scales were used to assess the psychosocial adjustment of the adolescents.

#### 1) Self-efficacy Scale

Self-efficacy was measured using a scale developed by Cha [66] and modified by Kim [67] consisting of three subscales: self-confidence, self-regulation efficacy, and task difficulty preference. Overall, the scale comprises 24 items rated on a 6-point scale. The total internal consistency was .87, and the internal consistency values were .82 for self-confidence, .93 for self-regulation efficacy, and .58 for task difficulty preference.

#### 2) Interpersonal efficacy scale

Paulhus [68] developed the Spheres-of-control battery items to measure personal efficacy and perceived control by categorizing them into individual, interpersonal, and sociopolitical scales. The Korean version measures interpersonal efficacy with a total of 10 items assessed on a 4-point scale [69]. Overall, the internal consistency of this scale was .73 in this study.

#### 3) The School Adjustment Scale

We used two subscales of the School Adjustment Scale developed by Lee and Kim [70], including school friends (friend relationship and cooperation, 10 items) and school teachers (teacher preference and intimacy, 10 items). These subscales assess interpersonal adjustment using a 4-point scale. The internal consistency values were .89 for the school friends subscale and .93 for the school teachers subscale.

#### Data analysis

For this study, a Pearson correlation analysis was performed using SPSS 21.0. The scores of each scale were standardized to account for differences in their scoring methods and, thus, accurately measure adolescent psychosocial adjustment. Next, to examine the structural relationships between maternal PRF, adolescent RF, and adolescent PSA, structural equation modeling (SEM) was conducted using Amos 21.0.

In terms of the three factors of the PRFQ-A (PM. CMS, IC) and the three factors of the RFQ-Y (U/C, Certainty, I/C), the purpose of this study was to examine the individual influence of these variables as latent variables in the model. Therefore, item parceling was conducted to consider each variable as a single factor and generate corresponding measurement variables. As an item parceling method, we used item-to-construct balance through a factorial algorithm [71]. Maximum likelihood was used to estimate the parameters in the structural equation, and the two-step approach proposed by Anderson and Gerbing [72] was applied for the validation of the measurement model and structural model. For the fit indices of the models, we used the Comparative Fit Index (CFI), Tucker Lewis Index (TLI), Root Mean Square Error of Approximation (RMSEA), and Standardized Root Mean Square Residual (SRMR), which consider the simplicity of the model and the sample size for both measurement and structural models, to evaluate the goodness of fit of the model. TLI and CFI values of .90 or higher indicate a good model [46]. RMSEA values of 0.06 or less indicate a ’good’ model fit, while values between 0.06 and 0.08 are considered ’acceptable’ or ’reasonable.’ However, values close to 0.08 should be interpreted with caution as they are on the borderline. RMSEA values of 0.10 or more indicate a ’poor’ model fit. SRMR values of 0.08 or less indicate a ’good’ fit, and lower values suggest a model that better fits the data [47]. Finally, the bootstrapping method [73] was used to test the statistical significance of the individual mediating effects of the three factors of the RFQ-Y on the relationship between the three factors of the maternal PRFQ-A and adolescent PSA identified in the structural model test.

### Results

#### Relationships between the PRFQ-A, RFQ-Y, and psychosocial adjustment

Maternal PM was positively associated with Maternal CMS, adolescents’ U/C, Certainty, and I/C, and negatively associated with PSA (S12 Table). Next, Maternal CMS was positively associated with adolescents’ Certainty, I/C, and PSA. Moreover, Maternal IC was positively correlated with Maternal CMS, adolescents’ U/C, Certainty, I/C, and PSA. Adolescents’ U/C was positively correlated with I/C and negatively correlated with PSA. Adolescents’ Certainty was positively correlated with I/C and PSA. Finally, adolescents’ I/C was positively correlated with PSA.

#### Structural model validation

**Fig 3:**
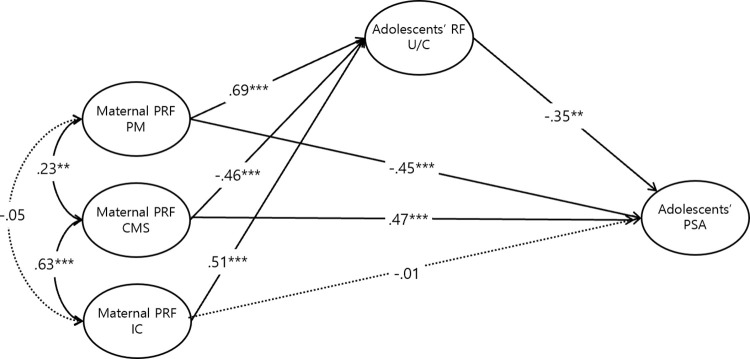
Standardized path coefficient of the adolescents’ U/C model. *p < .05, **p < .01, ***p < .001.

**Fig 4:**
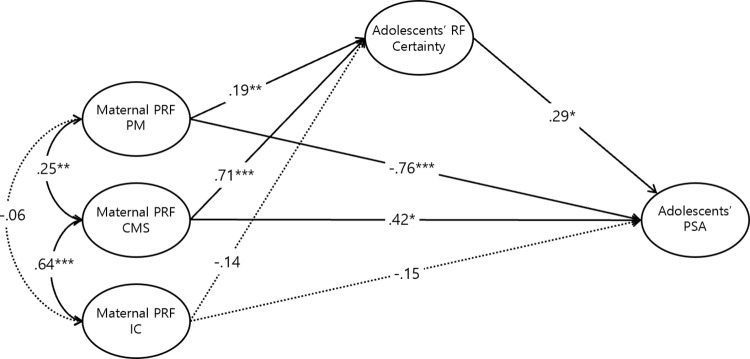
Standardized path coefficient of the adolescents’ certainty model. *p < .05, **p < .01, ***p < .001.

**Fig 5:**
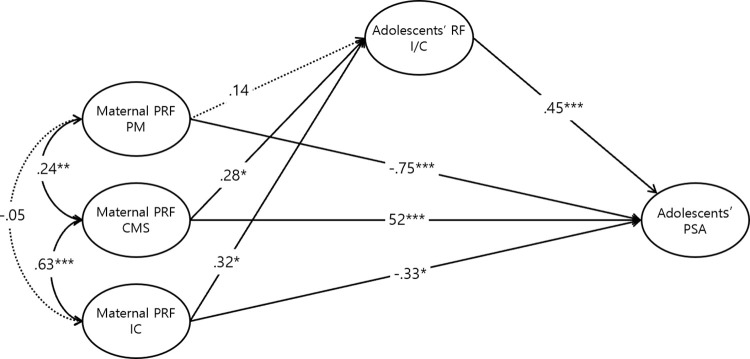
Standardized path coefficient of the adolescents’ I/C model. *p < .05, **p < .01, ***p < .001.

Path analysis was used to test the relationships between the study variables proposed in the null hypothesis model. The goodness-of-fit indices for all three structural models were found to be good. For the U/C model, the CFI was .946, TLI was .923, and RMSEA was .089 (90% confidence interval: .070-.109), which was somewhat high, but the SRMR met the criterion at .0754 [Fig 3]. The Certainty model had a CFI of .952, TLI of .932, RMSEA of .083 (90% confidence interval: .063-.103), and SRMR of .0713 [Fig 4]. Similarly, the I/C model had a CFI of .941, TLI of .917, RMSEA of .085 (90% confidence interval: .065-.105), and SRMR of .0767 [Fig 5]. Overall, these results indicate that all models met the criteria for good model fit.

#### Testing for mediation effect significance

Bootstrapping revealed statistically significant indirect effects in the U/C and Certainty models (S13 Table). Specifically, maternal PM, CMS, and IC significantly impacted adolescent PSA through U/C. Similarly, significant pathways were observed for PM and CMS through Certainty. However, no significant indirect effects were found in the I/C model.

### Discussion

In Study 2, we examined the mediating effect of adolescent RF on the relationship between maternal PRF and adolescent PSA. To do so, we conducted three structural model analyses with each RFQY subfactor as a mediator.

The structural model analyses revealed that consistent with the research hypotheses, maternal PM increased adolescents’ U/C and negatively influenced adolescents’ PSA. This finding is consistent with studies showing that mothers’ low RF negatively influences their children’s adaptive abilities, such as emotional and behavioral problems [74], and their children’s development of RF [75]. On the other hand, PM had a positive effect on adolescents’ Certainty, and Certainty was positively correlated with PSA, which may be explained by the mixed nature of the Certainty factor.

Maternal CMS reduced adolescents’ U/C and had a positive effect on PSA, partially mediated by adolescents’ Certainty. Contrary to expectations, maternal IC was not significantly related to adolescents’ PSA but increased adolescents’ U/C. It also had no significant effect on adolescents’ Certainty and PSA. In the adolescents’ IC model, maternal IC had a negative effect on adolescents’ PSA but a positive effect on adolescents’ IC. However, the mediation effect was not significant, and only the direct path was significant. The mixed results for the effects of maternal IC are discussed alongside the effects of maternal CMS on their children.

While maternal IC may show positive interest and curiosity in their children, it may also increase uncertainty and confusion. Luyten et al. [29] suggested that IC may be associated with intrusive parenting attitudes, implying that children might feel limited in their autonomy amidst parental expectations and interference, thereby reducing their self-assurance.

A confident attitude towards children is often linked to parental monitoring, where parents express understanding and interest in their children’s behavior, including their social interactions and daily activities [76]. This confident approach is closely related to parents’ certainty about their children’s mental states, as it reflects a clear and engaged understanding of their children’s thoughts and emotions. When parents are supportive and involved in their children’s lives, it fosters emotional security and social competence. Studies of Korean adolescents have demonstrated that parental monitoring positively impacts mental health and psychosocial adjustment [77–79], making adolescents feel more cared for and loved [80].

Research has shown cultural differences in parenting styles, such as how East Asian American children exhibit higher intrinsic motivation when mothers make minor decisions for them, while European American children show higher motivation when they make their own choices [81]. This pattern is consistent across different studies, including a comparative study of Chinese, Indian, and European-American college students [82]. These studies suggest that allowing children to make their own choices isn’t always necessary for fostering intrinsic motivation and that cultural context plays a significant role. For Korean adolescents, parental influence remains crucial, especially in contexts centered around academics and entrance exams.

Given this context, Korean adolescents may value their parents’ clarity and empathy towards their state of mind more than mere interest and curiosity. While IC can positively engage children with their inner states, it may also contribute to uncertainty and confusion when they are required to explore and make decisions independently. However, this interpretation is limited by the current findings, as this study only examined the association between parents’ and adolescents’ RFs. Therefore, future research that includes children’s perceptions of PRFs could provide more robust insights into the impact of PRFs on children.

The results show that adolescents’ U/C has a negative effect on adolescent PSA, consistent with previous studies that have demonstrated U/C to be associated with failure to mentalize, primarily in relation to negative traits. Adolescents’ Certainty and I/C were positively related to adolescent PSA in this work. Notably, in previous studies, certainty has been associated with mental health and personality pathology traits at low levels, but certainty was positively related to adolescent PSA in Study 2. These conflicting findings may reflect a mix of individuals in the Certainty factor, including people who understand the state of mind of others and those who think they do without sufficient reflection.

Consequently, the factor of Certainty, which reflects hypermentalizing, must be studied further, as this trait has also been found to be significantly related to borderline personality traits [9, 10]. For example, if respondents showing high levels of the factor Certainty share different characteristics, it may be worth considering developing additional items to distinguish between the two features or employing a measurement method such as midpoint scoring. Furthermore, studies involving clinical populations are needed to clarify the characteristics of the scale further.

The positive effect of adolescents’ I/C on PSA supports Duval et al.’s findings [11] that this scale indicates good reflective functioning. Given that I/C in the RFQ-Y focuses primarily on the state of mind of others, the I/C factor may be beneficial for interpersonal and social adjustment in a collectivistic society. However, the fact that the I/C factor was associated with difficulties in recognizing one’s emotions in Studies 1–2 suggests further exploration is required into the characteristics of Korean adolescents in relation to I/C. For example, studies are needed to determine how Korean adolescents experience the process of interest in the state of mind of themselves and others and, consequently, how they function in this context.

In summary, this study found that maternal PRF, specifically CMS and PM, had significant effects on adolescent RF and psychosocial adjustment. Maternal PM was associated with increased adolescent U/C and had a negative effect on PSA, whereas CMS decreased U/C and had a positive effect on PSA, partially mediated by adolescent certainty. Maternal IC had mixed effects, with a positive effect on adolescent IC but increased U/C and no significant effect on PSA. These findings highlight the complex and multidimensional nature of the PRF and underscore the need for further research to better understand the impact of different aspects of the PRF on adolescent development.

## General discussion and conclusion

The present study aimed to examine the relationship between parental and adolescent reflective function. The results of Study 1 confirm the applicability of the Korean versions of the PRFQ-A and RFQ-Y (See S1 and S2 Appendices for the full validated scales). Within each scale, the three dimensions measured shared some characteristics, but each dimension also showed independent features. These results highlight the need to consider the multidimensional aspects of RF. Therefore, it is necessary to continue research into the characteristics and effects of each factor related to RF.

In Study 2, the influence of maternal reflective functions on adolescents’ RF and psychosocial adjustment was found to be significant. The identified negative effects of low levels of maternal RF on adolescents’ psychosocial adjustments are consistent with findings from previous studies [83, 84]. Low levels of maternal RF could be regarded as a target for intervention to promote dysfunctional parent-child relationships adaptively. In addition, certain characteristics of maternal PRF that positively influenced adolescents’ PSA were identified. These results suggest that parent education can lay the foundation for improving parents’ overall PRF by clarifying the characteristics of positive PRF to parents and providing training in adaptive PRF. While further research is needed, this approach may have practical implications for enhancing parent-child relationships. It is important to note that our study did not include mental health history data for parents and children, which could have provided deeper insights. This limitation, due to the challenges of collecting sensitive information online, should be addressed in future research.

The findings also suggest that cultural context must be considered when understanding and applying the concept of RF. This study interpreted the unexpected results revealed in the RFQ-Y’s interest/curiosity factor in terms of the collectivist culture of Korea. Indeed, in such cultures, being concerned with the minds of others is related to trying to fit in with others and the group, and this behavior may be perceived as an adaptive trait in a collectivistic society. However, if there is insufficient self-reflection, an individual’s state of mind, including their feelings, desires, and intentions, could become undesirably repressed. Overall, these findings provide insights into the features of adaptive RF and how it can be fostered within cultural contexts.

The present study also found that “maternal certainty about mental status” had a more positive effect on adolescents’ RF and adjustment than “maternal interest and curiosity.” This result may reflect the nature of parent-child relationships in Korea, where parental influence is important until early adulthood. However, these interpretations should be considered cautiously, and further studies examining cultural differences are necessary to support this hypothesis.

In future studies, given the importance of paternal parenting and its impact on RF [24, 25], validation studies of the PRFQ-A that include fathers are recommended. In addition, participants of different age groups are needed to explore the characteristics of RF and the parent-child relationship at different developmental stages. Furthermore, differential parental influences have previously been found depending on the child’s gender, and the effects of parental non-mentalization have been reported to differ by gender and child age [85]. Therefore, further studies should examine differences in PRF across child age and gender. In addition, given that RF is closely related to various psychopathologies, particularly personality disorders, further studies involving clinical populations are needed.

Finally, one of the aims of this study was to validate an easy-to-use self-report measure and to explore the nature of related variables. However, additional methods, such as interviews and experimental studies that can measure RF should be included in future studies to clarify the nature of the measure and variables examined here. For example, a study using the Movie for the Assessment of Social Cognition (MASC), a computerized video task developed to assess mentalizing ability [86], can be conducted in conjunction with self-report measures. The limitations of the measure, as discussed in each study, should be considered when using it, and further research is required to refine the measure.

## Supporting information

S1 Table Descriptive statistical analysis results for K-PRFQ-A Sample 1. (DOCX)

S2 Table Descriptive statistical analysis results for K-PRFQ-A Sample 2. (DOCX)

S3 Table EFA results for the K-PRFQ-A. (DOCX)

S4 Table Correlations among the K-PRFQ-A subscales, mentalization, and empathy. (DOCX)

S5 Table Correlations among the K-PRFQ-A subscales, attachment, and distress. (DOCX)

S6 Table Correlations between the K-PRFQ-A subscales and parenting. (DOCX)

S7 Table Descriptive statistical analysis results for K-RFQ-Y Sample 1. (DOCX)

S8 Table Descriptive statistical analysis results for K-RFQ-Y Sample 2. (DOCX)

S9 Table EFA results for the K-RFQ-Y. (DOCX)

S10 Table Correlations among the K-RFQ-Y subscales, mentalization, and empathy. (DOCX)

S11 Table Correlations among the K-RFQ-Y subscales, internalizing/ externalizing problems, borderline traits, and narcissism traits. (DOCX)

S12 Table Correlations among the K-PRFQ-Y subscales, RFQ-Y subscales, and psychosocial adjustment. (DOCX)

S13 Table Bootstrapping results of the mediation effect. (DOCX)

S1 Appendix Korean Version of the Parental Reflective Functioning Questionnaire for Adolescents(K-PRFQ-A). (DOCX)

S2 Appendix Korean Version of Reflective Functioning Questionnaire for Youth(K-RFQ-Y). (DOCX)
