# Supplementary material for: Influences of maternal reflective functioning on adolescents’ psychosocial adjustment: The mediating role of adolescent’s reflective functioning
Source: PLoS One. 2024 Dec 26;19(12):e0312350. doi: 10.1371/journal.pone.0312350 (PMC11671003; doi:10.1371/journal.pone.0312350)
Supplement: S1 Table — (DOCX) [file pone.0312350.s001.docx]

**S1 Table: Descriptive Statistical Analysis Results for K-PRFQ-A Sample 1**

<*N*=203>

|  | M | SD | Range | Skewness | Kurtosis |
| --- | --- | --- | --- | --- | --- |
| PRFQ01 | 4.62 | 1.68 | 6 | -.43 | -.73 |
| PRFQ02 | 4.65 | 1.18 | 6 | -.44 | .10 |
| PRFQ03 | 5.05 | 1.07 | 5 | -.55 | .69 |
| PRFQ04 | 2.54 | 1.70 | 6 | 1.04 | .10 |
| PRFQ05 | 4.12 | 1.30 | 6 | -.23 | -.58 |
| PRFQ06 | 5.37 | 1.08 | 6 | -.64 | 1.07 |
| PRFQ07 | 3.69 | 1.36 | 6 | .18 | -.27 |
| PRFQ08 | 4.24 | 1.22 | 6 | -.31 | -.15 |
| PRFQ09 | 4.94 | 1.08 | 5 | -.21 | -.04 |
| PRFQ10 | 3.45 | 1.69 | 6 | .12 | -1.03 |
| PRFQ11 | 3.56 | 1.14 | 6 | .23 | .35 |
| PRFQ12 | 5.07 | .99 | 6 | -.48 | .95 |
| PRFQ13 | 3.13 | 1.57 | 6 | .31 | -.98 |
| PRFQ14 | 4.22 | 1.22 | 6 | -.40 | -.11 |
| PRFQ15 | 5.02 | 1.08 | 6 | -.79 | 1.26 |
| PRFQ16 | 3.47 | 1.55 | 6 | .10 | -.71 |
| PRFQ17 | 4.46 | 1.19 | 6 | -.36 | .16 |
| PRFQ18 | 4.91 | 1.57 | 5 | -.23 | -1.07 |
