# Supplementary material for: Influences of maternal reflective functioning on adolescents’ psychosocial adjustment: The mediating role of adolescent’s reflective functioning
Source: PLoS One. 2024 Dec 26;19(12):e0312350. doi: 10.1371/journal.pone.0312350 (PMC11671003; doi:10.1371/journal.pone.0312350)
Supplement: S2 Table — (DOCX) [file pone.0312350.s002.docx]

**S2 Table: Descriptive Statistical Analysis Results for K-PRFQ-A Sample 2**

<*N*=201>

|  | M | SD | Range | Skewness | Kurtosis |
| --- | --- | --- | --- | --- | --- |
| PRFQ01 | 4.48 | 1.62 | 6 | -.42 | -.71 |
| PRFQ02 | 4.74 | 1.19 | 6 | -.06 | -.07 |
| PRFQ03 | 5.01 | 1.04 | 5 | -.20 | -.04 |
| PRFQ04 | 2.75 | 1.75 | 6 | .69 | -.77 |
| PRFQ05 | 4.29 | 1.25 | 6 | -.19 | -.34 |
| PRFQ06 | 5.40 | .95 | 4 | -.06 | -.21 |
| PRFQ07 | 3.46 | 1.50 | 6 | .15 | -.60 |
| PRFQ08 | 4.43 | 1.16 | 5 | -.37 | -.16 |
| PRFQ09 | 4.97 | 1.07 | 5 | -.08 | .25 |
| PRFQ10 | 3.30 | 1.73 | 6 | .10 | -1.20 |
| PRFQ11 | 4.30 | 1.20 | 6 | -.51 | .23 |
| PRFQ12 | 5.08 | 1.03 | 5 | -.26 | .18 |
| PRFQ13 | 2.99 | 1.56 | 6 | .56 | -.44 |
| PRFQ14 | 4.34 | 1.20 | 6 | -.14 | -.13 |
| PRFQ15 | 4.94 | 1.11 | 5 | -.23 | .21 |
| PRFQ16 | 3.27 | 1.58 | 6 | .32 | -.72 |
| PRFQ17 | 4.47 | 1.18 | 6 | -.17 | .05 |
| PRFQ18 | 3.01 | 1.58 | 6 | .35 | -.88 |
