# Supplementary material for: Influences of maternal reflective functioning on adolescents’ psychosocial adjustment: The mediating role of adolescent’s reflective functioning
Source: PLoS One. 2024 Dec 26;19(12):e0312350. doi: 10.1371/journal.pone.0312350 (PMC11671003; doi:10.1371/journal.pone.0312350)
Supplement: S3 Table — (DOCX) [file pone.0312350.s003.docx]

**S3 Table: EFA Results for the K-PRFQ-A**

|  |  | Factor | | |
| --- | --- | --- | --- | --- |
|  |  | 1 | 2 | 3 |
| 16 | Often, my child’s behavior is too confusing to bother figuring out. | **.854** | -.103 | .029 |
| 13 | When my child is being difficult he or she does that just to annoy me. | **.811** | .094 | -.107 |
| 4 | My child cries or acts up/is difficult around strangers to embarrass me. | **.790** | .237 | -.055 |
| 10 | My child sometimes gets ill to keep me from doing what I want to do. | **.781** | .076 | .006 |
| 18 | I believe there is no point in trying to guess what my child feels. | **-.756** | -.171 | .160 |
| 7 | I find it difficult to empathize with the fantasies of my son/daughter. | **.715** | -.173 | .023 |
| 5 | I can completely read my child’s mind. | .018 | **.916** | -.130 |
| 2 | I always know what my child wants. | .051 | **.735** | .066 |
| 8 | I can always predict what my child will do. | -.084 | **.732** | .119 |
| 14 | I always know why I do what I do to my child. | .024 | **.682** | .099 |
| 17 | I always know why my child acts the way he or she does. | .035 | **.681** | .103 |
| 11 | I can sometimes misunderstand the reactions of my child. | **-.597** | **.415** | -.328 |
| 9 | I am often curious to find out how my child feels. | .073 | .160 | **.667** |
| 6 | I wonder a lot about what my child is thinking and feeling. | -.039 | -.107 | **.629** |
| 12 | I try to see situations through the eyes of my child. | -.121 | .112 | **.620** |
| 3 | I like to think about the reasons behind the way my child behaves and feels. | .003 | .282 | **.603** |
| 15 | I try to understand the reasons why my child misbehaves. | .008 | .015 | **.576** |
| Initial Eigenvalue (eigen value) | | 4.888 | 4.360 | 1.764 |
| Initial variance (%) | | 28.751 | 25.648 | 10.374 |
| Initial cumulative rate(%) | | 28.751 | 54.399 | 64.773 |
| KMO= .867  Bartlet's test( *x*² =1867.646, *df*=136 p<.001) | | | | |
