# Supplementary material for: Influences of maternal reflective functioning on adolescents’ psychosocial adjustment: The mediating role of adolescent’s reflective functioning
Source: PLoS One. 2024 Dec 26;19(12):e0312350. doi: 10.1371/journal.pone.0312350 (PMC11671003; doi:10.1371/journal.pone.0312350)
Supplement: S4 Table — (DOCX) [file pone.0312350.s004.docx]

**S4 Table: Correlations among the K-PRFQ-A subscales and mentalization and empathy**

|  | 1 | 2 | 3 | 4 | 4-1 | 4-2 | 4-3 | 4-4 | 5-1 |
| --- | --- | --- | --- | --- | --- | --- | --- | --- | --- |
| 1. pre-mentalizing modes | - |  |  |  |  |  |  |  |  |
| 2. Certainty about mental states | .09 |  |  |  |  |  |  |  |  |
| 3. interest and curiosity | -.10 | .54^***^ |  |  |  |  |  |  |  |
| 4. MZQ total | .62^***^ | -.08 | -.06 |  |  |  |  |  |  |
| 4-1. psychic equivalence mode | .52^***^ | -.02 | -.01 | .90^***^ |  |  |  |  |  |
| 4-2. refusing self-reflection | .65^***^ | -.08 | -.16^*^ | .92^***^ | .74^***^ |  |  |  |  |
| 4-3. emotional awareness | .41^***^ | -.13 | ^.08^ | .77^***^ | .61^***^ | .63^***^ |  |  |  |
| 4-4. regulation of affect | .50^***^ | -.08 | -.03 | .81^***^ | .67^***^ | .66^***^ | .54^**^ |  |  |
| 5-1. empathic concern | -.46^***^ | .16^*^ | .37^***^ | -.38^***^ | -.26^***^ | -.44^***^ | -.27^**^ | -.28^***^ |  |
| 5-2. perspective taking | -.36^***^ | .19^**^ | .34^***^ | -.35^***^ | -.30^***^ | -.39^***^ | -.21^**^ | -.21^**^ | .66^***^ |

^*^*p*<.05, ^**^*p*<.01, ^***^*p*<.001.
