# Supplementary material for: Influences of maternal reflective functioning on adolescents’ psychosocial adjustment: The mediating role of adolescent’s reflective functioning
Source: PLoS One. 2024 Dec 26;19(12):e0312350. doi: 10.1371/journal.pone.0312350 (PMC11671003; doi:10.1371/journal.pone.0312350)
Supplement: S5 Table — (DOCX) [file pone.0312350.s005.docx]

**S5 Table : Correlations among the K-PRFQ-A subscales and attachment and distress**

|  | Attachment Avoidance | Attachment Anxiety | Depression | Anxiety |
| --- | --- | --- | --- | --- |
| pre-mentalizing modes | .23^**^ | .69^***^ | .51^***^ | .42^***^ |
| Certainty about mental states | -.20^**^ | .06 | -.06 | -.07 |
| interest and curiosity | -.40^***^ | -.02 | -.07 | -.06 |

^*^*p*<.05, ^**^*p*<.01, ^***^*p*<.001.
