# Supplementary material for: Influences of maternal reflective functioning on adolescents’ psychosocial adjustment: The mediating role of adolescent’s reflective functioning
Source: PLoS One. 2024 Dec 26;19(12):e0312350. doi: 10.1371/journal.pone.0312350 (PMC11671003; doi:10.1371/journal.pone.0312350)
Supplement: S6 Table — (DOCX) [file pone.0312350.s006.docx]

**S6 Table: Correlations between the K-PRFQ-A subscales and parenting**

|  | PSCQ  warmth | PSCQ  rejection | PSCQ  structure | PSCQ  chaos | PSCQ  autonomy support | PSCQ  coercion | PSOC |
| --- | --- | --- | --- | --- | --- | --- | --- |
| pre-mentalizing modes | -.39^***^ | .66^***^ | -.01 | .68^**^ | -.15^*^ | .62^***^ | -.47^***^ |
| Certainty about mental states | .41^***^ | -.21^**^ | .10 | -.08 | .22^**^ | -.15^*^ | .42^***^ |
| interest and curiosity | .50^***^ | -.17^*^ | .23^**^ | -.16^*^ | .28^**^ | -.19^**^ | .39^**^ |

^*^*p*<.05, ^**^*p*<.01, ^***^*p*<.001.
