# Supplementary material for: Influences of maternal reflective functioning on adolescents’ psychosocial adjustment: The mediating role of adolescent’s reflective functioning
Source: PLoS One. 2024 Dec 26;19(12):e0312350. doi: 10.1371/journal.pone.0312350 (PMC11671003; doi:10.1371/journal.pone.0312350)
Supplement: S7 Table — (DOCX) [file pone.0312350.s007.docx]

**S7 Table: Descriptive Statistical Analysis Results for K-RFQ-Y Sample 1**

<*N*=257>

|  | M | SD | Range | Skewness | Kurtosis |
| --- | --- | --- | --- | --- | --- |
| RFQY01 | 4.09 | 1.14 | 5 | -.78 | .52 |
| RFQY02 | 3.01 | 1.05 | 5 | .25 | -.06 |
| RFQY03 | 4.17 | 1.09 | 5 | -.63 | .75 |
| RFQY04 | 3.68 | 1.34 | 5 | -.24 | -.68 |
| RFQY05 | 4.47 | 1.17 | 5 | -.71 | .73 |
| RFQY06 | 3.66 | 1.22 | 5 | -.02 | -.78 |
| RFQY07 | 3.76 | 1.09 | 5 | -.44 | .05 |
| RFQY08 | 3.88 | 1.13 | 5 | -.52 | -.02 |
| RFQY09 | 4.10 | .98 | 5 | -.38 | .43 |
| RFQY10 | 4.22 | .96 | 5 | -.46 | .65 |
| RFQY11 | 3.50 | 1.29 | 5 | -.13 | -.91 |
| RFQY12 | 3.35 | 1.22 | 5 | -.03 | -.70 |
| RFQY13 | 3.69 | 1.08 | 5 | -.27 | -.28 |
| RFQY14 | 3.60 | 1.15 | 5 | -.24 | -.46 |
| RFQY15 | 3.74 | 1.18 | 5 | -.32 | -.50 |
| RFQY16 | 3.51 | 1.13 | 5 | -.08 | -.45 |
| RFQY17 | 3.79 | 1.04 | 5 | -.33 | .31 |
| RFQY18 | 3.58 | 1.21 | 5 | -.37 | -.69 |
| RFQY19 | 3.54 | 1.25 | 5 | -.17 | -.71 |
| RFQY20 | 3.74 | 1.07 | 5 | -.36 | -.32 |
| RFQY21 | 3.49 | 1.17 | 5 | -.10 | -.71 |
| RFQY22 | 3.75 | .99 | 5 | -.44 | -.80 |
| RFQY23 | 4.01 | 1.14 | 5 | -.52 | .39 |
| RFQY24 | 4.11 | 1.01 | 5 | -.78 | 1.21 |
| RFQY25 | 3.80 | 1.06 | 5 | -.41 | -.06 |
