# Supplementary material for: Influences of maternal reflective functioning on adolescents’ psychosocial adjustment: The mediating role of adolescent’s reflective functioning
Source: PLoS One. 2024 Dec 26;19(12):e0312350. doi: 10.1371/journal.pone.0312350 (PMC11671003; doi:10.1371/journal.pone.0312350)
Supplement: S8 Table — (DOCX) [file pone.0312350.s008.docx]

**S8 Table: Descriptive Statistical Analysis Results for K-RFQ-Y Sample 2**

<*N*=239>

|  | M | SD | Range | Skewness | Kurtosis |
| --- | --- | --- | --- | --- | --- |
| RFQY01 | 4.05 | .95 | 5 | -.56 | .57 |
| RFQY02 | 3.94 | .92 | 5 | -.30 | -.14 |
| RFQY03 | 4.08 | .92 | 5 | -.37 | .61 |
| RFQY04 | 3.78 | 1.11 | 5 | -.18 | -.25 |
| RFQY05 | 4.41 | .98 | 5 | -.51 | .88 |
| RFQY06 | 3.61 | 1.02 | 5 | -.21 | -.03 |
| RFQY07 | 3.68 | .93 | 5 | -.07 | -.09 |
| RFQY08 | 3.90 | .89 | 5 | -.24 | .39 |
| RFQY09 | 4.02 | .86 | 5 | -.56 | 1.22 |
| RFQY10 | 4.21 | .88 | 5 | -.28 | 1.04 |
| RFQY11 | 3.72 | 1.06 | 5 | -.33 | -.10 |
| RFQY12 | 3.62 | 1.07 | 5 | .01 | -.11 |
| RFQY13 | 3.59 | .90 | 5 | -.05 | .01 |
| RFQY14 | 3.65 | .99 | 5 | -.23 | .02 |
| RFQY15 | 3.80 | 1.01 | 5 | -.44 | -.08 |
| RFQY16 | 3.58 | .91 | 5 | .16 | .02 |
| RFQY17 | 3.89 | .89 | 5 | -.20 | .34 |
| RFQY18 | 3.63 | .88 | 5 | -.38 | .30 |
| RFQY19 | 3.49 | 1.07 | 5 | -.28 | .13 |
| RFQY20 | 3.63 | .99 | 5 | -.01 | .20 |
| RFQY21 | 3.64 | .97 | 5 | -.39 | .37 |
| RFQY22 | 3.61 | .91 | 5 | -.18 | -.11 |
| RFQY23 | 3.87 | .94 | 5 | -.13 | -.01 |
| RFQY24 | 3.97 | .87 | 5 | .03 | .22 |
| RFQY25 | 3.69 | .91 | 5 | -.09 | -.24 |
