# Supplementary material for: Influences of maternal reflective functioning on adolescents’ psychosocial adjustment: The mediating role of adolescent’s reflective functioning
Source: PLoS One. 2024 Dec 26;19(12):e0312350. doi: 10.1371/journal.pone.0312350 (PMC11671003; doi:10.1371/journal.pone.0312350)
Supplement: S9 Table — (DOCX) [file pone.0312350.s009.docx]

**S9 Table: EFA Results for the K-RFQ-Y**

|  |  | Factor | | |
| --- | --- | --- | --- | --- |
|  |  | 1 | 2 | 3 |
| 11 | When I get angry I say things without really knowing why I am saying them. | **.698** | -.088 | .037 |
| 15 | When I get angry I say things that I later regret. | **.681** | .055 | -.030 |
| 12 | Those close to me often seem to find it difficult to understand why I do things. | **.677** | .047 | -.165 |
| 14 | Strong feelings often cloud my thinking. | **.608** | .182 | -.158 |
| 18 | If I feel unsure of myself, I can behave in ways that offend others. | **.601** | .048 | -.025 |
| 21 | Sometimes I find myself saying things and I have no idea why I said them. | **.598** | -.076 | .283 |
| 19 | Sometimes I do things without really knowing why. | **.587** | .025 | .144 |
| 6 | I believe other people are too confusing to bother figuring out. | **-.506** | .140 | -.073 |
| 22 | I can mostly predict what someone else will do. | -.037 | **.718** | .043 |
| 16 | My feelings about a person are hardly ever wrong. | .166 | **.682** | -.204 |
| 25 | I know exactly what my close friends are thinking. | -.061 | **.679** | .055 |
| 13 | I usually know exactly what other people are thinking. | .024 | **.676** | .055 |
| 7 | I am a good mind reader. | -.102 | **.672** | .127 |
| 20 | I can tell how someone is feeling by looking at their eyes. | .062 | **.613** | .040 |
| 5 | I believe that people can see a situation very differently based on their own beliefs and experiences. | .008 | -.121 | **.622** |
| 10 | Understanding the reasons for people’s actions helps me to forgive them. | -.019 | .093 | **.609** |
| 3 | I feel that, if I am not careful, I could get in the way of another person’s life. | .153 | -.103 | **.572** |
| 23 | I’m often curious about the meaning behind others’ actions. | .097 | .103 | **.561** |
| 24 | I pay attention to the impact of my actions on others’ feelings. | -.069 | .148 | **.514** |
| 09 | In an argument, I keep the other person’s point of view in mind. | -.145 | .138 | **.509** |
| Initial Eigenvalue (eigen value) | | 5.241 | 3.098 | 1.793 |
| Initial variance (%) | | 26.204 | 15.490 | 8.965 |
| Initial cumulative rate(%) | | 26.204 | 41.694 | 50.660 |
| KMO=.846, Bartlet's test(x² = 1851.122, df=210 p<.001) | | | | |
