# Supplementary material for: Influences of maternal reflective functioning on adolescents’ psychosocial adjustment: The mediating role of adolescent’s reflective functioning
Source: PLoS One. 2024 Dec 26;19(12):e0312350. doi: 10.1371/journal.pone.0312350 (PMC11671003; doi:10.1371/journal.pone.0312350)
Supplement: S10 Table — (DOCX) [file pone.0312350.s010.docx]

**S10 Table: Correlations among the K-RFQY subscales and mentalization and empathy**

|  | 1 | 2 | 3 | 4 | 4-1 | 4-2 | 4-3 | 4-4 |
| --- | --- | --- | --- | --- | --- | --- | --- | --- |
| 1.Uncertainty/Confusion | - |  |  |  |  |  |  |  |
| 2. certainty | .32*** |  |  |  |  |  |  |  |
| 3. Intertest/Curiosity | .20** | .40*** |  |  |  |  |  |  |
| 4. MZQ total | .64*** | .12 | .21** |  |  |  |  |  |
| 4-1. psychic equivalence mode | .56*** | .11 | .24*** | .86** |  |  |  |  |
| 4-2. refusing self-reflection | .53*** | .14* | .10 | .87** | .59** |  |  |  |
| 4-3. emotional awareness | .46*** | .09 | .30*** | .79** | .58** | .59** |  |  |
| 4-4. regulation of affect | .59*** | .02 | .08 | .78** | .60** | .56** | .58** |  |
| 5. Empathy | .03 | .08 | .20** | .01 | .04 | .00 | -.01 | -.03 |

^*^*p*<.05, ^**^*p*<.01, ^***^*p*<.001.
