# Supplementary material for: Influences of maternal reflective functioning on adolescents’ psychosocial adjustment: The mediating role of adolescent’s reflective functioning
Source: PLoS One. 2024 Dec 26;19(12):e0312350. doi: 10.1371/journal.pone.0312350 (PMC11671003; doi:10.1371/journal.pone.0312350)
Supplement: S11 Table — (DOCX) [file pone.0312350.s011.docx]

**S11 Table: Correlations among the K-RFQY subscales internalizing/externalizing problems and borderline traits and narcissism traits.**

|  | Depression | Anxiety | behavioral control difficulties | emotional control difficulties | BPD traits | Grandiose Narcissism | Vulnerable Narcissism |
| --- | --- | --- | --- | --- | --- | --- | --- |
| 1.Uncertainty/Confusion | .34^***^ | .37^***^ | .50^***^ | .46^***^ | .51^***^ | .43^***^ | .56^***^ |
| 2. certainty | .21^**^ | .22^**^ | .22^**^ | .16^*^ | .33^***^ | .26^***^ | .25^***^ |
| 3. Intertest/Curiosity | .08 | .15^*^ | -.05 | .07 | .13^*^ | .26^***^ | .16^*^ |

^*^*p*<.05, ^**^*p*<.01, ^***^*p*<.001.
