# Supplementary material for: Influences of maternal reflective functioning on adolescents’ psychosocial adjustment: The mediating role of adolescent’s reflective functioning
Source: PLoS One. 2024 Dec 26;19(12):e0312350. doi: 10.1371/journal.pone.0312350 (PMC11671003; doi:10.1371/journal.pone.0312350)
Supplement: S12 Table — (DOCX) [file pone.0312350.s012.docx]

**S12 Table: Correlations among the K-PRFQ-Y subscales and RFQY subscales and psychosocial adjustment**

|  | 1 | 2 | 3 | 4 | 5 | 6 |
| --- | --- | --- | --- | --- | --- | --- |
| 1. Mother's pre-mentalizing | - |  |  |  |  |  |
| 2. mother's Certainty | .24^**^ | - |  |  |  |  |
| 3. mother's interest/curiosity | -.04 | .52^***^ | - |  |  |  |
| 4. adolescent Uncertainty/Confusion | .52^***^ | .05 | 15^*^ | - |  |  |
| 5. adolescent certainty | .18^*^ | .60^***^ | .23^**^ | .14 | - |  |
| 6. adolescent Intertest/Curiosity | .35^***^ | .43^***^ | .38^***^ | .25^**^ | .56^***^ | - |
| 7. adolescent psychosocial adjustment | -.38^***^ | .29^***^ | .20^**^ | -.45^***^ | .24^**^ | .35^***^ |

^*^*p*<.05, ^**^*p*<.01, ^***^*p*<.001.
