# Supplementary material for: Influences of maternal reflective functioning on adolescents’ psychosocial adjustment: The mediating role of adolescent’s reflective functioning
Source: PLoS One. 2024 Dec 26;19(12):e0312350. doi: 10.1371/journal.pone.0312350 (PMC11671003; doi:10.1371/journal.pone.0312350)
Supplement: S13 Table — (DOCX) [file pone.0312350.s013.docx]

**S13 Table: Bootstrapping results of the mediation effect**

| Pathway | B Value | ***SE*** | 95% confidence interval  (Bias-corrected bootstrap) | |
| --- | --- | --- | --- | --- |
|  |  |  | Lower | Upper |
| PM -> U/C -> PSA | -.110 | .054 | -.223 | -.018 |
| CMS -> U/C -> PSA | .098 | .058 | .027 | .283 |
| IC -> U/C -> PSA | -.169 | .113 | -.519 | -.036 |
| PM -> Certainty -> PSA | .025 | .019 | .001 | .075 |
| CMS -> Certainty -> PSA | .128 | .077 | .008 | .309 |
| IC -> Certainty -> PSA | -.036 | .051 | -.210 | .019 |
| PM -> I/C -> PSA | .029 | .024 | -.002 | .096 |
| CMS -> I/C -> PSA | .078 | .055 | -.015 | .196 |
| IC -> I/C -> PSA | .131 | .118 | -.008 | .497 |
