# Supplementary material for: Influences of maternal reflective functioning on adolescents’ psychosocial adjustment: The mediating role of adolescent’s reflective functioning
Source: PLoS One. 2024 Dec 26;19(12):e0312350. doi: 10.1371/journal.pone.0312350 (PMC11671003; doi:10.1371/journal.pone.0312350)
Supplement: S1 Appendix — (DOCX) [file pone.0312350.s014.docx]

**S14 Appendix. Korean Version of the Parental Reflective Functioning Questionnaire for Adolescents(K-PRFQ-A)**

Listed below are a number of statements concerning you and your child. Please read each item carefully and decide whether you agree or disagree and to what extent.

Use the following rating scale, with 7 if you strongly agree, and 1 if you strongly disagree; the midpoint, if you are neutral or undecided, is 4.

| 1 | 2 | 3 | 4 | 5 | 6 | 7 |
| --- | --- | --- | --- | --- | --- | --- |
| Strongly Disagree |  |  |  |  |  | Strongly  Agree |

2. I always know what my child wants.

3. I like to think about the reasons behind the way my child behaves and feels.

4. My child cries or acts up/is difficult around strangers to embarrass me.

5. I can completely read my child’s mind.

6. I wonder a lot about what my child is thinking and feeling.

7. I find it difficult to empathize with the fantasies of my son/daughter.

8. I can always predict what my child will do.

9. I am often curious to find out how my child feels.

10. My child sometimes gets ill to keep me from doing what I want to do.

12. I try to see situations through the eyes of my child.

13. When my child is being difficult he or she does that just to annoy me.

14. I always know why I do what I do to my child.

15. I try to understand the reasons why my child misbehaves.

16. Often, my child’s behavior is too confusing to bother figuring out.

17. I always know why my child acts the way he or she does.

18. I believe there is no point in trying to guess what my child feels.
