# Supplementary material for: Influences of maternal reflective functioning on adolescents’ psychosocial adjustment: The mediating role of adolescent’s reflective functioning
Source: PLoS One. 2024 Dec 26;19(12):e0312350. doi: 10.1371/journal.pone.0312350 (PMC11671003; doi:10.1371/journal.pone.0312350)
Supplement: S2 Appendix — (DOCX) [file pone.0312350.s015.docx]

**S15 Appendix. Korean Version of Reflective Functioning Questionnaire for Youth**

**(K-RFQ-Y)**

Read each of the following statements and decide the one response you feel most clearly describes you. Don't think too much about it. Your first response is usually the best.

| 1 | 2 | 3 | 4 | 5 | 6 |
| --- | --- | --- | --- | --- | --- |
| Strongly  Disagree | Disagree | Disagree  Somewhat | Agree  Somewhat | Agree | Strongly  Agree |

3. I feel that, if I am not careful, I could get in the way of another person’s life.

5. I believe that people can see a situation very differently based on their own beliefs and experiences.

6. I believe other people are too confusing to bother figuring out.

7. I am a good mind reader.

9. In an argument, I keep the other person’s point of view in mind.

10. Understanding the reasons for people’s actions helps me to forgive them.

11. When I get angry I say things without really knowing why I am saying them.

12. Those close to me often seem to find it difficult to understand why I do things.

13. I usually know exactly what other people are thinking.

14. Strong feelings often cloud my thinking.

15. When I get angry I say things that I later regret.

16. My feelings about a person are hardly ever wrong.

18. If I feel unsure of myself, I can behave in ways that offend others.

19. Sometimes I do things without really knowing why.

20. I can tell how someone is feeling by looking at their eyes.

21. Sometimes I find myself saying things and I have no idea why I said them.

22. I can mostly predict what someone else will do.

23. I’m often curious about the meaning behind others’ actions.

24. I pay attention to the impact of my actions on others’ feelings.

25. I know exactly what my close friends are thinking.
